# Drug versus placebo randomized controlled trials in neonates: A review of ClinicalTrials.gov registry

**DOI:** 10.1371/journal.pone.0171760

**Published:** 2017-02-13

**Authors:** Emilie Desselas, Claudia Pansieri, Stephanie Leroux, Maurizio Bonati, Evelyne Jacqz-Aigrain

**Affiliations:** 1 Department of Pediatric Pharmacology and Pharmacogenetics, Hopital Robert Debré, Paris, France; 2 Clinical Investigation Center INSERM CIC1426, Hopital Robert Debré, Paris, France; 3 Department of Public Health, Laboratory for Mother and Child Health, IRCCS-Istituto di Ricerche Farmacologiche “Mario Negri”, Milan, Italy; 4 Université Paris 7 Diderot, Paris, France; University of Nottingham, UNITED KINGDOM

## Abstract

**Background:**

Despite specific initiatives and identified needs, most neonatal drugs are still used off-label, with variable dosage administrations and schedules. In high risk preterm and term neonates, drug evaluation is challenging and randomized controlled trials (RCT) are difficult to conduct and even more is the use of a placebo, required in the absence of a reference validated drug to be used as comparator.

**Methods:**

We analyzed the complete ClinicalTrials.gov registry 1) to describe neonatal RCT involving a placebo, 2) to report on the medical context and ethical aspects of placebo use.

**Results:**

Placebo versus drug RCT (n = 146), either prevention trials (n = 57, 39%) or therapeutic interventions (n = 89, 61%), represent more than a third of neonatal trials registered in the National Institute of Health clinical trial database (USA) since 1999. They mainly concerned preterm infants, evaluating complications of prematurity. Most trials were conducted in the USA, were single centered, and funded by non-profit organizations. For the three top drug trials evaluating steroids (n = 13, 9.6%), erythropoietin (EPO, n = 10, 6.8%) and nitric oxide (NO, n = 9, 6.2%), the objectives of the trial and follow-up were analyzed in more details.

**Conclusion:**

Although a matter of debate, the use of placebo should be promoted in neonates to evaluate a potential new treatment, in the absence of reference drug. Analysis of the trials evaluating steroids showed that long-term follow-up of exposed patients, although required by international guidelines, is frequently missing and should be planned to collect additional information and optimize drug evaluation in these high-risk patients.

## Introduction

Neonates are highly vulnerable compared to older children and adults: preterm and term neonates are characterized by different degrees of physiological immaturity, they develop specific diseases, need adapted drug formulations and dosages, have different responses to drugs and specific risks of adverse events [1–3]. Accordingly, specific drug evaluation is required in neonates and protocols should include short and long term safety studies.

Despite specific initiatives and identified needs [4–6], more than 90% of neonatal drugs are still used unlicensed or off-labeled, with variable dosage administrations and schedules [7–10].

According to the Food and Drug Administration (FDA) and European Medicine Agency (EMA) pediatric decision tree, drug evaluation in pediatrics, including in neonates, has to be optimized by analyzing all available preclinical and clinical data in adults and children, by adapting drug evaluation to diseases specificity related to prematurity, to developmental differences in drug disposition and effects between neonatal age groups, and by considering all ethical issues [11–13]. In addition, the opportunity to use an adaptive trial design that is potentially able to reduce the number of patients to be included in drug trials should be considered, although not frequently used in neonatology until now. [14,15]

Randomized controlled trials (RCT) remain the gold standard for drug evaluation [16,17] but they are challenging and even more is the use of a placebo, required in the absence of a reference validated drug to be used as comparator.

During our previous report on neonatal drug trials registered in the ClinicalTrials.gov database, the number of clinical trials using a placebo appeared surprisingly high [18].Therefore, we further analyzed the studies during which a placebo was administered in order to report on the medical context and ethical aspects of placebo use in neonates.

## Methods

### Search strategy

We analyzed the complete ClinicalTrials.gov registry between its launch in 1999 and December 31^st^, 2015. A total of 206 629 records of clinical trials from more than 100 countries were registered. We searched for all records that involved neonates: the registry categorizes age at which participants are eligible for enrollment as child (≤ 17 years), adult (18 to 65 years) and senior (≥ 66 years). To select the records of interest, we used "neonates and drugs" as search words in the free text section and only phases I to IV were selected.

### Study selection

Records were analysed individually, evaluated for relevance and duplicates were removed. The initial selection was made by one researcher (ED), deletions or classification were all confirmed by a second researcher (EJA). The characteristics of neonatal recruitment were selected and analysed for all trials that included term and preterm neonates. Only placebo RCT based on study analysis were selected. Therefore, trials were not included if they were not limited to neonates, were not randomised or did not have a placebo arm.

### Data extraction

Data were extracted, using a standard extraction form validated previously [18] including study settings, year of beginning and estimated duration, location and number of participating centers, recruitment status (ongoing, completed and suspended), study design, number of participants and characteristics of the population (term, preterm), condition under study, trial phase, drug under testing and comparison treatments, main purpose of study (prevention or curative treatment), previous publication title, and indication of drug use.

The primary sponsor was classified as governmental (NIH, US federal and governments from non-US countries), industry, and non-profit organizations (including clinical research networks, research associations, hospitals, universities, foundations and others).

Drugs were coded according to the Anatomical Therapeutic and Chemical (ATC) classification [19]

Data was extracted from information included in the database. Terms as placebo, prevention and treatment data were individually analyzed after the first extraction, based on ClinicalTrials.gov definition of terms. Placebo is defined as a substance that does not contain active ingredients and is made to be physically indistinguishable (that looks and tastes identical) from the actual drug being studied. Treatment purpose is defined as a protocol designed to evaluate one or more interventions for treating a disease, syndrome or condition. Prevention is defined as a protocol designed to assess one or more interventions aimed at preventing the development of a specific disease or health condition.

### Statistical analysis

To facilitate the analysis a database was subsequently designed, and Statistical analysis was done using Excel program. Results are given in number, mean and standard deviation and percentage.

## Results

### General overview of neonatal placebo/drug trials

**Fig 1:**
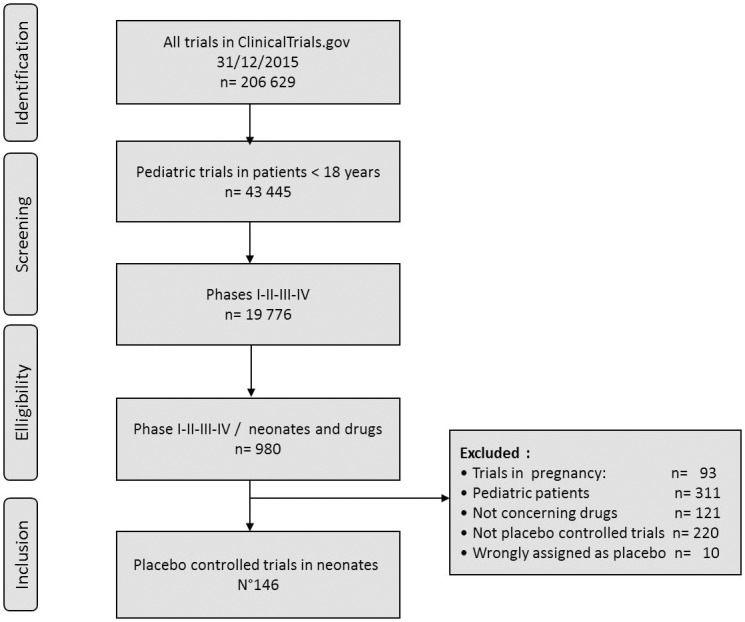
Flow chart of ClinicalTrials.gov registry analysis.

Among all (n = 206 629) drug trials registered in ClinicalTrials.gov since 1999, 423 were neonatal therapeutic trials, 146 were drug versus placebo trials involving neonates and represented 34.4% of all therapeutic trials on neonates (Fig 1), 16 and 22 were registered before 2000 and 2005 respectively, 45 between 2006–2010 and 61 between 2011–2015.

All trials were randomized, blinded (n = 144/146, 98%), double (n = 123, 84.2%) or triple arms (n = 23, 15.8%) and classified phases 1 and 1/2 to phase 4. The number of patients to be included was 299 ± 296 (mean ± SD, range: 7–14035 n = 142) but most trials planned to include less than 100 patients (n = 77, 52.7%). Among the 146 trials, 91 (62.3%) were completed, 21 (14.4%) were still recruiting, 34 (23.3%) were either not recruiting or with unknown status. “Table 1”

**Table 1:** Characteristics of placebo randomized controlled trials registered in ClinicalTrials.gov (1999–2015).

	N	%
**TOTAL NUMBER OF TRIALS**	146	100.0
**NEONATES STUDIED**		
Preterm	70	47.9
Term	35	24.0
Preterm and Term	41	28.1
**NUMBER OF PATIENTS PLANNED**		
0–50	35	24.6
51 to 100	42	29.5
>100	65	45.7
unknown	4	0.2
**SPONSOR (N)**		
Industry	21	14.4
Government*	22	15.0
Non-federal/Non-profit^1^	81	55.5
Government+Industry	2	1.4
Non-federal/Non-profit+ Industry	9	6.2
Non-federal/Non-profit+Government	10	6.8
Non-federal+Industry+Governement	1	0.7
**STUDY PHASES**		
Phases 1 and 1/2	21	14.4
Phases 2 and 2/3	57	39.0
Phase 3	40	27.4
Phase 4	28	19.2
**BLINDING**		
Blinded	144	98.6
Open label	2	1.4
**LOCATION (N)**		
*SINGLE CENTER*	**81**	**55.5**
Africa	2	2.5
Middle East Asia	22	27.2
Europe	18	22.2
North America	35	43.2
South America	4	4.9
Oceania	0	0
*MULTICENTER NATIONAL TRIALS*	**43**	**29.5**
Europa	4	9.3
North America	38	88.4
Asia	1	2.3
*MULTICENTER INTERNATIONAL TRIALS*	**10**	**6.8**
Africa	1	10.0
Middle East Asia	3	30.0
Europe	10	100.0
North America	5	50.0
South America	1	10.0
Oceania	2	20.0
*UNKNOWN*	**12**	**8.2**
**RECRUITMENT STATUS**^2^		
** Open—recruiting**	21	14.4
Open—not recruiting	15	10.3
Completed	91	62.3
Unknown	19	13.0
**LENGHT OF STUDY (as planned—years)**		
<1	40	27.4
1–2	56	38.4
3–4	25	17.1
>4	25	17.1
Unknown	0	0.0
PUBLICATION*^2^		
Yes	58	39.7
Patients reported in the publication versus planned in the trial		
± 10% of planned	35	24.0
More than 10% higher	15	10.2
More than 10% lower	8	5.5
No	88	60.3

* GOVERNMENT = all the Governmental Institutions + US Federal Agency + NIH (National Institute of Health)

^1^ NON-FEDERAL = University+ Organization+ Hospital+ Clinical Research Network

^2^ Updated in December 2017

Trials involving a placebo were conducted with two distinct neonatal strategies: a preventive strategy was evaluated in 57 trials (39.0%), and a therapeutic strategy with evaluation of a “new drug” or a “new indication” in 89 curative trials (61.0%), following different study designs (Table 2). In addition, a few studies were safety studies or modifications of dosages schedules (n = 17, 11.4%).

**Table 2:** Classification of the placebo–controlled drug trials in neonates in ClinicalTrials.gov (1999–2015) according to design and aim.

Strategy	Total	Curative Treatment	Disease Prevention
	146	89	57
**Design**	**N = 146**		
Placebo versus drug parallel assignement	**105**	58	47
Placebo versus drug trial as add-on therapy	**36**	25	11
Factorial design	**3**	3	0
Cross-over	**2**	2	0
**Aim**	**N = 146**		
Efficacy—Safety	**117**	64	53
Efficacy with rescue treatment	**12**	11	1
Pharmacokinetics	**17**	15	2

### Neonatal groups and aims

Thirty-five (24.0%) of the trials included exclusively term newborns while 70 (47.9%) and 41 (28.1%) included premature newborns or both preterm and term newborns respectively. Altogether, preterm were present in 111 (76.0%) studies.

**Fig 2:**
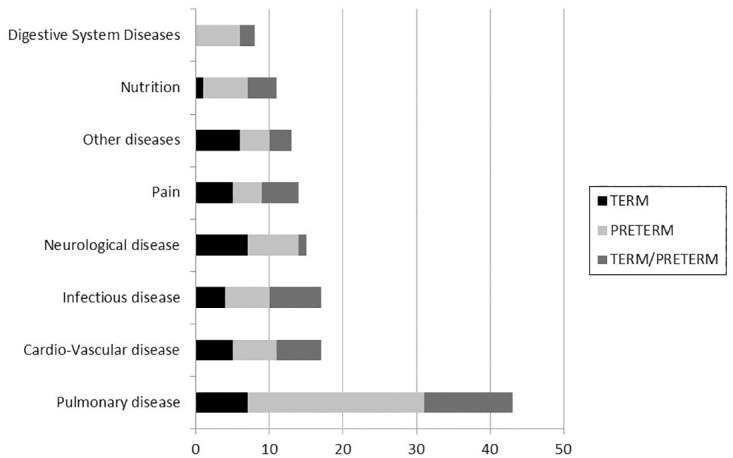
Differences in the diseases and conditions between preterm and term neonates in the placebo—drug trials registered in ClinicalTrials.gov.

Diseases and conditions were different between preterm and term neonates (Fig 2).

**Fig 3:**
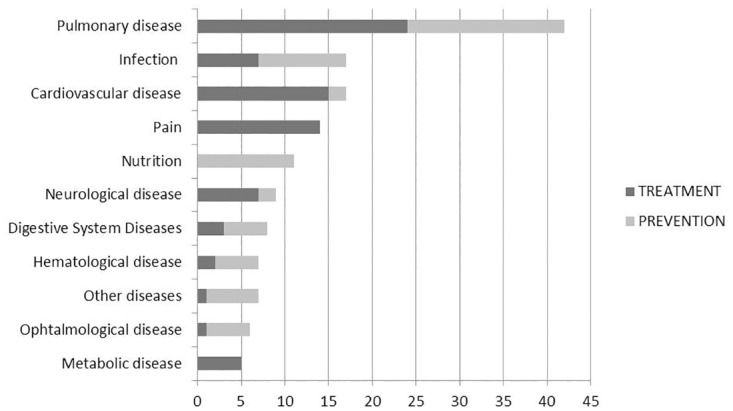
Differences in neonatal diseases and conditions evaluated in the prevention or treatment placebo—drug trials registered in ClinicalTrials.gov.

Most trials focused on prevention or treatment of complications of prematurity: bronchopulmonary dysplasia (BPD), enterocolitis, viral and fungal infections, retinopathy or anemia of prematurity (Fig 3).

**Fig 4:**
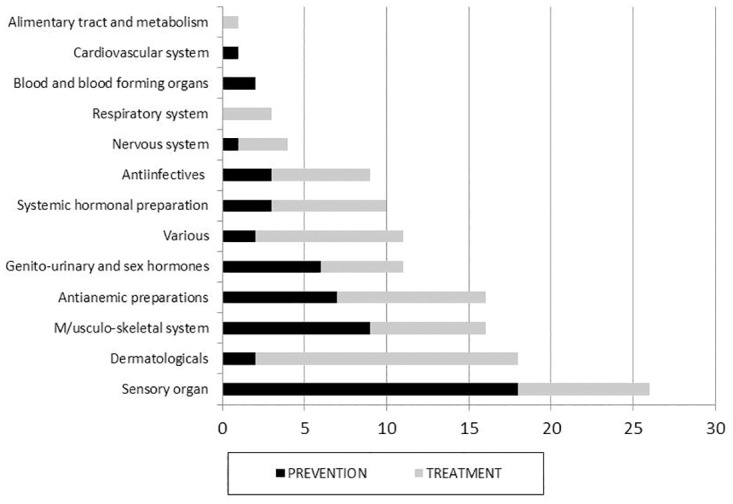
ATC classification of the drugs evaluated in the neonatal placebo-drug trials in ClinicalTrials.gov.

Eighty-three different drugs were under evaluation. ATC classification of the drugs evaluated in these trials is presented in Fig 4.

The primary aim of the trials was either treatment (n = 89, 61.0%) or disease prevention (n = 57, 39.0%). In both groups, design was predominantly placebo drug parallel assignment (n = 105, 72.0%) or add-on therapy (n = 36, 24.7%).

Both prevention and treatment trials were predominantly efficacy and safety trials with parallel assignment between two arms. A total of 36 (24.7%) trials were add-on studies in which all patients received the experimental drug or the placebo in addition to the currently used treatment. Examples include addition of CC10 or lucinactant to surfactant in respiratory distress syndrome, addition of Nitric Oxide to standard of care in respiratory failure, EMLA alone or in addition to glucose to reduce pain during venipuncture (Table 2). Beside clinical efficacy, 3 placebo-drug trials evaluated a biological endpoint in clinically asymptomatic patients and in 4 studies: after the first phase of recommended duration, patients were randomized into two arms and in the first arm, drug treatment was continued while in the second arm, a placebo was administered ».

Three arm interventions had pharmacokinetics as a primary or secondary endpoint (n = 16, 11.0%) with a placebo arm and arms comparing different doses, or had two treatment arms (reference drug and drug under evaluation) and one placebo arm (n = 8, 5.5%).

### Geographical repartition and sponsorship

Most placebo trials took place in North America (n = 78, 53.4%), mainly in the USA and were single center (n = 81, 55.5%).

The multicenter trials were predominantly national trials, conducted in a total of 359 centers. They were predominantly conducted in North America (centers: n = 203, 56.5%), Europe (n = 52, 14.5%) and Asia (n = 48, 13.4%). Only 24 (5.3%) of the multicenter trials were international, mainly in Europe (n = 10) and in the United States (n = 5).

The number of neonatal placebo trial registrations was limited in the late 90’s (from 0–3 per year worldwide) but increased in 2002 (1–2 trials per year) mostly in the USA and the first trials were registered after 2004 in Europe and Asia.

The number of trials involving industry or government as only source of funding were 33 (22.6%) and 22 (15.4%) respectively, but governments with or without non-profit organizations funded at least partially, the majority of the trials (n = 113, 77.4%), both single and multicenter. Sponsors were predominantly non-profit organizations (n = 81, 55.5%), and “multiple funding” were less frequent (n = 22, 15.1%) (Table 1)

### Analysis of the top drugs (or lecture of drugs)

The three top drugs were steroids (n = 13, 9.6%), erythropoietin (EPO) (n = 10, 6.8%) and nitric oxide (NO, n = 9, 6.2%).

We analyzed in more details the trials evaluating steroids and NO in prevention of BPD, as this indication is specific to the neonatal age group.

Five different steroid drugs were examined: hydrocortisone (n = 6), methylprednisone (n = 1), inhaled budesonide (n = 4), beclomethasone (n = 1), dexamethasone (n = 1). Some of these trials are completed, some are still recruiting or active but not recruiting. Their objectives were disease prevention or treatment of BPD, acute lung injury or transient tachypnea. Their primary objective was in all cases to compare the short-term effects of the drug versus placebo on respiratory function, cardiovascular or neurological effects. Key publications are available for 8 of the 10 completed trials. (Table 3). [20–27]

**Table 3:** Detailed analysis of randomized-controlled trials evaluating steroids in neonates.

N	Start / End of study (years) *	Title / Primary outcome / Long-term follow-up	Trial status** Study Chair (SC) or Principal Investigator (PI) (Publications)
**RCT hydrocortisone versus placebo**			
1	2001/2005 Prevention trial NCT00004669	Phase II Pilot Study of Early Cortisol Replacement to Prevent Bronchopulmonary Dysplasia PO: Estimate (versus placebo) the efficacy of cortisol replacement therapy during the first 12 days of life for prevention of bronchopulmonary dysplasia. LT: no	Completed SC: KL Watterberg [20]
2	2005/2015 Prevention trial lNCT00167544	Randomized Trial of Hydrocortisone in Very Preterm High-Risk Infants PO: Total Cerebral Volume as Measured by Volumetric Brain MRI LT: no	Completed PI: NA Parikh [21]
3	2006/2011 Treatment trial NCT00358748	Early Use of Hydrocortisone in Hypotensive Very Low Birth Weight Infants. PO: Total cumulative dose of dopamine at 48 hours of study drug administration and by day 7 of life in neonates with gestational age ≤ 30 weeks LT: no	Completed PI: H Osiovich [22]
4	2007/2013 Treatment trial NCT00590018	Corticosteroids (hydrocortisone) in postoperative critically ill Neonates With Low Cardiac Output syndrome With Congenital Heart Disease PO: HR, BP, mVO2 to assess cardiac output in patients aged <1month LT: no	Completed PI: HA Dickerson
5	2008/2016 Prevention trial NCT 00623740	PREMILOC: Trial to Prevent Bronchopulmonary Dysplasia in Very Preterm Neonates PO: survival without BPD at 36 weeks PMA gestational age, in neonates between 24 weeks and 27 weeks + 6 days gestational age LT: neurodevelopmental outcome at 18 months to 3 years	Completed PI: O Baud [23]
6	2014/2018 Treatment trial NCT01954056	Hydrocortisone for Term Hypotension PO: Death or neurodevelopmental impairment 34 Weeks’ gestational age and older LT: Neurodevelopmental Impairment [Birth to 22–26 month corrected age]	Active—Not recruiting SC: E.Fernandez
**RCT Budesonide versus placebo**			
7	2009/ 2013 Prevention trial NCT00883532	Prevention of Chronic Lung Disease (CLD) in Preterm Infants PO: Chronic lung disease morbidity among the survival at 36 postconceptional weeks LT: Neurodevelopment at 2 years of age	Completed PI: TF Yeh [24]
8	2010 /2016 Treatment trial NCT01035190	Efficacy and Safety of Inhaled Budesonide in Very Preterm Infants at Risk for Bronchopulmonary Dysplasia PO: To determine whether inhalation of Budesonide within 12 hours of life improves survival without BPD at 36 weeks GA in infants born between 23 and 27 weeks GA LT: neurodevelopment at a corrected age of 18–22 months	Completed PI: D Bassler [25]
10	2012 / 2015 Treatment trial NCT01858129	Inhaled Corticosteroids for the Treatment of Transient Tachypnea (TTN) of the Newborn PO: assessment of respiratory distress at 48 hours, reflected by TTN clinical score in Infants Born at >34 Weeks Gestation LT: no	Recruiting PI: A Kugelman
9	2016 / 2018 Prevention trial NCT01895075	Inhaled budesonide in non-ventilated infants at high risk of bronchopulmonary dysplasia: the i-BUD pilot study. PO: Total days on supplemental oxygen from birth to discharge LT: no	Not yet recruiting PI: M Dunn
**RCT of other corticosteroids versus placebo**			
11	1992 / 1994 Treatment trial NCT00011362	Dexamethasone Therapy in VLBW Infants at Risk of CLD PO: Number of days from randomization to ventilator independence LT: Morbidity and mortality from respiratory causes during the first year (12 months of age)	Completed PI: LA Papille [26]
12	1993/1999 Prevention trial NCT00000576	Inhaled Beclomethasone to Prevent Chronic Lung Disease PO: bronchopulmonary dysplasia at 28 days of age in premature infants (birth weight less than 1251 grams, gestational age less than 33 weeks, and postnatal age 3 to f14 days, under mechanical ventilation LT: no	Completed [27]
13	2014/2017 Treatment trial NCT01757899	Effects and Safety of Infusion of Low-Doses of Methylprednisolone in Early ALI and ARDS (Acute Lung Injury and Acute Respiratory Distress Syndrome) in patients up to 17 Years (PEDALI) PO: Effects on pulmonary organ function at 24 months of entry LT: Complications at 12 months of entry (safety issue)	Recruiting SC: MCM Barbosa

* estimated year,

** Definitions as provided in Clinicaltrial.gov: Completed: "last subject, last visit" has occurred, Terminated: the clinical study has stopped recruiting or enrolling participants early and will not start again, Participants are no longer being examined or treated.

PO: Primary Objective, LO: Long-term, PI: Principal Investigator, SC: Study Chair

We also analyzed in details the trials evaluating iNO versus placebo (nitrogen gas or oxygen). All but one of these trials had a short-term primary outcome evaluating death and BPD at 36 weeks’ post-menstrual age. The results were published in 5 cases, the other trials are completed or still recruiting (Table 4). [28–33]

**Table 4:** Detailed analysis of randomized-controlled trials evaluating iNO: Inhaled Nitric Oxide (iNO) in neonates.

N	Estimated dates of Start / End (years) *	Title / Primary outcome / Long-term follow-up	Trial status** Principal Investigator (PI) (Publications)
1	1995 / 1998 NCT00005776	Inhaled Nitric Oxide Study for Respiratory Failure in Newborns (NINOS) PO: Death or initiation of ECMO before hospital discharge or 120 days of life LT: outcome assessed at 18 to 24 mos of age.	Terminated PI: RA. Ehrenkranz [28]
2	2000/2006 NCT00000548	Inhaled NO in Prevention of Chronic Lung Disease PO: Survival without chronic lung disease (CLD) [Time Frame: 36 weeks] LT: Neurodevelopmental outcome through two years of age	Completed PI: Ballard R [29]
3	2001 / 2006 NCT00016523	Inhaled Nitric Oxide for Preterm Infants With Severe Respiratory Failure (Preemie iNO) PO: Death or Bronchopulmonary Dysplasia at 36 weeks post-conceptional age LT: Neurodevelopmental outcome at 18–22 months corrected age	Terminated PI: KP Van Meurs [30]
4	2002 / 2005 NCT00041548	Inhaled Nitric Oxide in Neonates With Elevated A-a DO2 (alveolar-arterial oxygen) Gradients Not Requiring Mechanical Ventilation (gestational age >34 completed weeks) PO: PaO2 level at baseline, then every hour for 6 hours LT: no	Terminated PI: Waldemar Carlo [31]
5	2005 /2016 NCT01220687	Safety and Efficacy Study of Nitric Oxide for Inhalation on Chronic Lung Disease in Premature Babies PO: Survival Without Bronchopulmonary Dysplasia (BPD) in Preterm Infants With Respiratory Distress at 36 weeks gestational age LT: no	Active, not recruiting PI: JCMercier [32]
6	2007 / 2016 NCT00955487	Examining the use of non-invasive inhaled nitic oxide to reduce chronic lung disease on premature newborns. PO: Combined endpoint of bronchopulmonary dysplasia and mortality [Time Frame: Week 36 or earlier, if participants are discharged from the hospital LT: Long term follow up at 1 and 2 years	Ongoing, but not recruiting PI: J Kinsella [33]
7	2008 / 2016 NCT00515281	Inhaled Nitric Oxide and Neuroprotection in Premature Infants PO: Bronchopulmonary dysplasia at 36 weeks of age corrected LT: Neurodevelopment at two years	Active—not recruiting PI: MD. Schreiber
8	2009/2011 NCT0092253*2*	**Inhaled Nitric Oxide (INO) In Hypoxic Respiratory Failure** PO: Arterial Blood Gases [Time Frame: Day 1 through Day 6] LT: no	*Withdrawn prior to enrollment*.
9	2009 / 2014 NCT00931632	Inhaled Nitric Oxide (INO) for the Prevention of Bronchopulmonary Dysplasia (BPD) in Preterm Infants PO: Survival Without BPD at 36 Weeks LT: no	Completed Study Director: J Baldassarre
10	2011 / 2016 NCT01220687	Inhaled Nitric Oxide (iNO) as an Adjunct to Neonatal Resuscitation PO: To investigate whether iNO decreases the supplemental oxygen exposure in the preterm infants who require continuous positive airway pressure (CPAP) or positive pressure ventilation (PPV) during resuscitation as per Neonatal Resuscitation Program (NRP) protocol. LT: no	Recruiting PI: Kris Sekar,
11	2013 / 2014 NCT01748045	Study of Inhaled Nitric Oxide and Respiratory Outcomes in Late Preterm Infants PO: Primary combined endpoint of alive without the need for intubation or mechanical ventilation within the first week of life LT: no	Terminated PI: Jennifer W Lee
12	2016 / 2017 NCT01891500	Early iNO for Oxidative Stress, Vascular Tone and Inflammation in Babies With Hypoxic Respiratory Failure (gestational Gestational age ≥ 35 weeks gestation) PO: Biomarkers of oxidative injury. LT: no	Not yet open for recruitment PI: C Bazacliu

* estimated year

** Definitions as provided in Clinicaltrial.gov: Completed: "last subject, last visit" has occurred, Terminated: the clinical study has stopped recruiting or enrolling participants early and will not start again, Participants are no longer being examined or treated.

PO: Primary Objective, LO: Long-term, PI: Principal Investigator

## Discussion

The present study was undertaken to quantify and discuss the use of a placebo in RCTs conducted in neonates. We identified 146 studies registered in ClinicalTrials.gov from 1999 to 2015. Most of them were prevention trials or add-on studies, while evaluation of therapeutic interventions were less frequent. They mainly concerned preterm infants, evaluating management of complications of prematurity. The majority of the trials were conducted in the USA, were single centered and funded by non-profit organizations, mostly hospitals and universities.

A placebo is defined by its lack of specific pharmacological or physiological efficacy for a patient’s condition. When administered during a RCT, accumulated evidence suggests that in the placebo arm, the observed effect is a genuine psychobiological event attributable to the overall therapeutic context. The overall response of the active treatment arm is the result of the treatment itself and the context in which it is given, quantified by the response to placebo, relying on complex neurobiologic mechanisms, influenced by psychosocial factors [34–37].

Our research focused on neonatal placebo versus drug trials, which represent one third of all neonatal trials and became more frequent after 2002, probably framed by the *International Ethical Guidelines for Biomedical Research Involving Human Subjects***,** ensuring the regulation for the use of placebo [38]. Whatever the medical situation and patients’ groups, the use of a placebo remains controversial and rises major ethical concerns both in clinical research and clinical practice [39–42]. It may be an option only if respecting the principle of clinical equipoise and respect of patients’ wellbeing. Therefore, it is important to carefully evaluate the balance between potential benefits and disadvantages of such design to discuss ethical issues [43–45]

According to our data, neonatal randomized placebo-controlled trials are conducted in the two different contexts of disease prevention or treatment: a prevention trial only aims at health benefit without inducing any harm while a treatment trial aims at improving health care, while limiting harm. Here again, the conduct of neonatal placebo trials remains a matter of debate and controversies with arguments «for and against» [6,46,47]. A placebo can only be used when no standard treatment for that disease exists; in other cases, the new drug should be compared with the gold-standard [48]. On one hand, defenders of placebo advocate that both specificity of neonatal diseases and the absence of reference treatment, ie validated comparator, are strong arguments to use a placebo. This is for example the case for a trial evaluating the efficacy of erythropoietin for neuroprotection in very preterm infants

In our research, the majority of trials were dealing with prematurity and specific related complications or concerned prevention or treatment of diseases occurring only in neonates. In such situations, the natural history and pathophysiology of diseases, developmental pharmacology data and identification and validation of relevant biomarkers are required [1,49] and it is difficult to extrapolate both efficacy and safety from data obtained in older children or adults as drugs are frequently used off label [7,50]. Consequently, uncertainty on neonatal efficacy and high risk of adverse events do exist when extrapolation to neonates is used, justifying specific drug evaluation compared to a placebo in the subgroup of pediatric patients [3] On the other hand, for obvious scientific and medical reasons, and even in the absence of a reference treatment, the use of a placebo cannot be envisaged in medical situations that might lead to a “loss of chance” for the patient. Indeed, many drugs enter the neonatal care arena because of proven efficacy in older pediatric patients or even adults, because clinicians perceive them to have a useful spectrum of activity compared to ‘older’ drugs or even in the absence of drug available in the therapeutic indication. In such cases, resorting to an off-label drug is frequent in neonatal care, while administrating a placebo would be questionable. In addition, although a placebo effect was observed in different pediatric studies and quantified as even higher that in adults [49–54], the placebo effect in neonates is not a consensual reality [55,56].

The debate on the use of placebo in neonatal trials requires a deep analysis of all ethical issues to promote and protect newborn health, here again with arguments “for or against” [48,57] For example, a placebo RCT that compares a drug administrated by intra-muscular injection should better be compared to a sham injection rather than an intra-muscular injection of placebo [58]. During our research, 15 placebo RCT targeted analgesics during painful procedures in neonates and 9 did not have any rescue therapy, although newborns do feel pain but even more, have a lower threshold of pain. In such situations, the use of a placebo should be questioned [59]. Indeed, placebo RCT for pain studies in infants might not be the best methodological approach to prove new pharmacological therapies. The “add-on” trial design where the drug under evaluation and the placebo are added to the “empiric therapy” has been used in many trials with pros and cons: among them, such design allows to maintain current therapy and define the effect of empiric therapy in the placebo arm but has additional safety risks

We also analyzed in more details the studies evaluating corticosteroids used for prevention or treatment of specific neonatal diseases (BPD, cardiovascular or neurological diseases), as the recent guidelines state that there are insufficient evidence to recommend both early or late administration [60,61] (S1 Table). However, important and recent data were obtained against placebo for prophylactic low-dose hydrocortisone, showing that in extremely preterm infants, the rate of survival without BPD at 36 weeks’ postmenstrual age was significantly increased, and for early inhaled budesonide showing a lower incidence of BPD at 36 weeks’ postmenstrual age. Although beneficial effects of both early and late corticosteroids were already identified, such placebo/drug RCT were justified by the absence of reference arm, demonstrating significant benefits for both drugs [62,63]. Benefits associated with early hydrocortisone may not overweight the risks of it use although long-term deleterious effects were mainly associated with dexamethasone and not hydrocortisone and potential undesired effects on neurodevelopment at preschool age should be evaluated in future long-term studies [64]

The 14 trials evaluating iNO were analysed in two Cochrane reviews [65,66], and 12 were included in a recent meta-analysis, 5/14 and 6/12 respectively being iNO versus placebo RCTs trials [67]. Neonates presenting with risks factors of deleterious outcome (gestational age, oxygenation index, pulmonary hypertension…) were enrolled and followed up to 36 weeks of age. Guidelines on the use of iNO were issued by the Canadian Paediatric Society and the American Academy of Pediatrics [68–70](S2 Table). Within the recent NIH Consensus Development Conference Statement, future research directions recommend the use of a placebo control when designing future randomized trials to assess optimal iNO treatment (timing, dose and duration), long-term safety (not a primary outcome in the ongoing trials analyzed here) and predictive markers of outcome (biomarkers, neuroimaging) [71].

Placebo trials in neonates are rare. A bias in data analysis might occur 1) if not all trials are reported, especially single site trials involving a relatively small number of infants, 2) if they are not all identified. Therefore, web-based registries are major tools to increase transparency in the conduct of clinical trials. We selected clinicaltrials.gov registry in our initial research [18] and in this complementary analysis, although it does not focus on pediatric trials. However, the registry the registry started more than 15 years ago, it is public and permits the registration of all clinical trials regardless the disease, age group, type of intervention or country, and therefore allows description of the current scope of pediatric and neonatal trials [72] The only pediatric database that we know of was DEC-net. (Drug Evaluation in Children-network), a web- based register of trials on drug therapy in children was supported by the European Commission in 2006: data collection was oriented to the paediatric population, with a clear definition of age groups. The first data analysis was made available in 2008, but the database closed at the end of the project [73,74]. Other registries or platforms have limitations when it comes to pediatric trials: the International Clinical Trials WHO Registry Platform (ICTRP) [75], the EU Clinical Trials Register [76] containing information on interventional clinical trials since 2004. However, to our knowledge, none of them has a focus on paediatric trials and the absence of identification of paediatric age groups does not to analyze the trials recruiting both neonates and other pediatric patients and to separate premature and term neonates.

In addition, the low number of industry-sponsored trials in neonates is of concern [77,78], although legislation was put in place in the USA and Europe to improve this situation [79–83] Following these major changes in the Regulatory framework of pediatric drug evaluation, the number of pediatric trials increased but the impact of the new regulation remains limited [84,85]. Unfortunately, the significant economic benefits to the pharmaceutical industry are not matched by the benefits for pediatric patients. In addition, elaboration of an undifferentiated placebo may be technically difficult and very expensive.

## Conclusion

In the clinical trial database setup by the National Institute of Health in the USA [81] placebo controlled RCT represent more than a third of neonatal trials. Although matter of large debates, a placebo is justified to evaluate efficacy of the potential new treatment when no reference drug is available Medical issues, scientific validity, methodological and ethical specificities are to be taken into account in this high risk patients [86] and all the available data should be analyzed to design and conduct such studies.

## Supporting information

S1 Table Guidelines on use of corticosteroids to prevent or treat broncho-pulmonary dysplasia in neonates. (DOCX)

S2 Table Guidelines on use of inhaled nitric oxide in neonates. (DOCX)

S1 PRISMA Checklist (DOCX)
