# Supplementary material for: Drug versus placebo randomized controlled trials in neonates: A review of ClinicalTrials.gov registry
Source: PLoS One. 2017 Feb 13;12(2):e0171760. doi: 10.1371/journal.pone.0171760 (PMC5305102; doi:10.1371/journal.pone.0171760)
Supplement: S1 Table — (DOCX) [file pone.0171760.s001.docx]

| **S1 table**  **Guidelines on use of corticosteroids to prevent or treat bronchopulmonary dysplasia in neonates** | | | |
| --- | --- | --- | --- |
| **Hydrocortisone** | | | |
|  | | **Prevention** | **Treatment** |
| **Country, Year [Ref]** | **Name of society/ title** | **Indications** | **Indications** |
| **Canada 2012 [60]** | **Canadian Paediatric Society Fetus and Newborn Committee**  [***Postnatal corticosteroids to treat or prevent chronic lung disease in preterm infants***](https://onlinereview.cps.ca/papers/postnatal-corticosteroids-chronic-lung-disease/print_ready.pdf) | * Postnatal corticosteroids within the first seven days of life is not recommended  * Early hydrocortisone treatment may be beneficial in a specific population of patients; however, there is insufficient evidence to recommend its use for all infants at risk of BPD | * Hydrocortisone is not recommended for treating BPD |
| **US 2010**  **Reaffirmed in**  **2014**  **[61]** | **American Academy of Pediatrics**  ***Postnatal corticosteroids to prevent or treat bronchopulmonary dysplasia*** | * Early low-dose hydrocortisone treatment may be used in specific population of patients such as infants exposed to inflammation in utero, however, insufficient evidence to recommend use for all infants at risk of BPD.  * Insufficient evidence to recommend high-dose hydrocortisone | * Early low-dose hydrocortisone treatment may be used in specific population of patients such as infants exposed to inflammation in utero, however, insufficient evidence to recommend use for all infants at risk of BPD.  * Insufficient evidence to recommend high-dose hydrocortisone |
