# Supplementary material for: Drug versus placebo randomized controlled trials in neonates: A review of ClinicalTrials.gov registry
Source: PLoS One. 2017 Feb 13;12(2):e0171760. doi: 10.1371/journal.pone.0171760 (PMC5305102; doi:10.1371/journal.pone.0171760)
Supplement: S2 Table — (DOCX) [file pone.0171760.s002.docx]

| **S2 Table**  **Guidelines on use of inhaled nitric oxide in neonates** | | | |
| --- | --- | --- | --- |
| **Inhaled Nitric Oxide** | | | |
|  | | **Prevention** | **Treatment** |
| **Country, Year [Ref]** | **Name of society/ title** | **Indications** | **Indications** |
| **Canada 2012**  **[68]** | **Canadian Paediatric Society, Fetus and Newborn Committee**  ***Inhaled nitric oxide use in newborns*** |  | Current evidence shows that iNO improves oxygenation and decreases the combined outcome of death or need for extracorporeal membrane oxygenation in infants >35 weeks’ gestational age at birth. Its role in managing preterm infants <35 weeks’ gestational age is not yet established. iNO is safe when administered in tertiary care settings using strict protocols and monitoring. |
| **US**  **2014**  **[69]** | **American Academy of Pediatrics.**  ***Use of Inhaled Nitric Oxide in Preterm Infants*** | * The results of randomized controlled trials, traditional meta-analyses, and an individualized patient data meta-analysis study indicate that neither rescue nor routine use of iNO improves survival in preterm infants with respiratory failure  * The preponderance of evidence does not support treating preterm infants who have respiratory failure with iNO for the purpose of preventing/ ameliorating BPD, severe intraventricular hemorrhage, or other neonatal morbidities  * The incidence of cerebral palsy, neurodevelopmental impairment, or cognitive impairment in preterm infants treated with iNO is similar to that of control infants | * The results of 1 multicenter, randomized controlled trial suggest that treatment with a high dose of iNO (20 ppm) beginning in the second postnatal week may providea small reduction in the rate of BPD. However, these results need to be confirmed by other trials.  * An individual-patient data meta-analysis that included 96% of preterm infants enrolled in all published iNO trials found no statistically significant differences in iNO effect according to any of the patient-level characteristics, including gestational age, race, oxygenation index, postnatal age at enrollment, evidence of pulmonary hypertension, and mode of ventilation.  * There are limited data and inconsistent results regarding the effects of iNO treatment on pulmonary outcomes of preterm infants in early childhood. |
| **US**  **2010**  **[70]** | **AHRQ Publication No. 11-E001**  ***Inhaled Nitric Oxide in Preterm Infants*** |  | There was a seven percent reduction in the risk of the composite outcome of death or BPD at 36 weeks PMA for infants treated with iNO compared to controls, but no reduction in death or BPD alone. There is currently no evidence to support the use of iNO in preterm infants with respiratory failure outside the context of rigorously conducted randomized clinical trial |
